# Serum Intact Parathyroid Hormone Level After Total Thyroidectomy or Total Thyroidectomy Plus Lymph Node Dissection for Thyroid Nodules: Report From 296 Surgical Cases

**DOI:** 10.5812/ijem.3462

**Published:** 2012-09-30

**Authors:** Yukiko Yano, Chie Masaki, Kiminori Sugino, Mitsuji Nagahama, Wataru Kitagawa, Hiroshi Sibuya, Koichi Ito

**Affiliations:** 1 Ito Hospital, Tokyo, Japan

**Keywords:** Hypoparathyroidism, Hypocalcemia, Thyroidectomy, Thyroid Nodule, Parathyroid

## Abstract

**Background:**

Transient hypocalcemia is one of the postoperative complications of thyroidectomy for thyroid nodules, and intraoperative and postoperative intact parathyroid hormone (iPTH) assays are used to predict postoperative hypocalcemia.

**Objectives:**

The current study was conducted to evaluate a single serum iPTH measurement on postoperative day 1 (POD 1) as a means to predict hypocalcemia occurrence after total thyroidectomy (TT).

**Patients and Methods:**

The subjects consisted of 36 patients who underwent TT and 260 patients who underwent TT plus lymph node (LN) dissection for thyroid nodules treatment. The TT performance procedure to prevent postoperative hypoparathyroidism combines parathyroid gland preservation in situ with autotransplantation of resected or devascularized parathyroid glands. The patients’ serum iPTH level was measured on POD 1, and their serum calcium level was measured on POD 1 and on POD 3 while they were still inpatients. The serum iPTH level was subequently measured at each outpatient clinic visit until it recovered to the normal range.

**Results:**

Hypoparathyroidism after TT and TT plus LN dissection was ultimately diagnosed in a total of 229 patients, and in 69 of them hypocalcemia was diagnosed on POD 1. All of the 69 patients diagnosed with hypocalcemia received calcium and vitamin D supplementation therapy. The serum iPTH level of 67 of 229 patients was within normal range on POD 1, and four of them developed hypocalcemia on POD 1. Permanent hypoparathyroidism developed in 37 of 296 patients after undergoing TT or TT plus LN dissection for thyroid nodules in the hospital.

**Conclusions:**

A single serum iPTH measurement on POD 1 is useful to determine whether or not to start calcium and vitamin D supplementation in order to maintain normocalcemia after surgery.

## 1. Background

Total thyroidectomy (TT) is accepted worldwide as the standard surgical procedure to managebenign bilateral nodular thyroid disease, and thyroid cancer. Hypocalcemia is one of the postoperative complications of thyroid surgery, and is caused by injury to the parathyroid glands during the operation (1, 2). Most series of studies have reported an incidence of around 20-30% and symptoms ranging from mild paresthesia and tingling to severe cramps, tetany, and convulsions. The traditional approach to detect postoperative hypocaclemia of inpatient clinic assessment, and to monitor serum calcium levels used by many institutions worldwide. Intact parathyroid hormone (iPTH) assay, intraoperative and postoperative, is used to predict postoperative hypocalcemia development following total thyroidectomy for various thyroid diseases (3). A strong correlation between postoperative serum iPTH levels and the incidence of hypocalcemia after total thyroidectomy has been reported. The postoperative blood specimen collections timing to measure serum iPTH in previous studies has varied (3).

A single serum iPTH level measurement in the early postoperative period is a simple examination, and patients within the normal range level, have a low risk of post-TT hypocalcemia. A retrospective review of the surgical and pathological findings records, and hypoparathyroidism management after surgery were performed in the current study.

## 2. Objectives

The present study aimed to assess the usefulness of a single serum iPTH measurement on postoperative day (POD) 1 as a means to predict hypocalcemia after TT. The serum calcium and iPTH levels of a group of patients who underwent TT plus neck lymph node (LN) dissection and a group of patients who underwent TT alone were compared. This study was conducted at the hospital, which is specialized in thyroid disease treatment, and about 1800 thyroid operations are performed there annually.

## 3. Patients and Methods

### 3.1. Subjects

The medical records of the all patients who had undergone TT in the hospital during January 2010 to August 2010 were reviewed, and patients who had undergone TT or TT plus LN dissection and been followed up for six months or more after surgery were selected for inclusion in this study. TT had been performed for thyroid nodules in 296 patients, and they had been followed up for six months or more after surgery. The histological diagnosis was papillary thyroid cancer in 255 patients, follicular thyroid cancer in 14 patients, follicular thyroid adenoma in 4 patients, and adenomatous nodules in 23 patients. Histopathological examination had revealed papillary thyroid cancer in five of the follicular thyroid cancer patients and one of the adenomatous nodule patients.

### 3.2. Methods

TT performance procedure to prevent postoperative hypoparathyridism combines preservation of parathyroid glands in situ with autotransplantation of resected or devascularized parathyroid glands. After removing the parathyroid glands they were minced until they had a gel-like consistency. The minced parathyroid tissue was autotransplanted into a pocket that had been created in the patient’s sternocleidomastoid muscle (4).

Serum total calcium (normal range, 8.0 to 10.5 mg/dL), albumin, and iPTH (normal range, 15.0 to 65.0 pg/mL) were measured by routine automated procedures. The calcium concentration was corrected by the formula: corrected calcium concentration = calcium concentration (mg/dL) + 4 – albumin (g/dL). A single serum iPTH measurement was made on POD 1. If the serum iPTH value on POD 1 was below the normal range, calcium supplementation and/or vitamin D supplementation was started. Patients who had no hypocalcemia symptoms, e.g., numbness, on POD 1 and patients whose serum calcium value was below the normal range on POD 1 received calcium and/or vitamin D supplementation. Patients who developed tetany due to hypocalcemia on the day of the operation received an infusion of glucose calcium. The doses prescribed for calcium supplementation and vitamin D supplementation to treat the patients who developed hypoparathyroidism were adjusted to maintain their serum calcium value within the normal range. All of the patients’ calcium level was measured on POD 3, and the dose prescribed for oral calcium supplementation was adjusted where necessary. The patients were discharged on POD 4, and were followed up by the endocrine surgeon in the outpatient clinic. The serum iPTH and calcium levels of the patients with postoperative hypoparathyroidism on POD 1 were measured at 1- to 3-month intervals during the follow-up period. The serum iPTH values of all patients with postoperative hypocalcemia on POD 1 were measured until they recovered to the normal range. Vitamin D and/or calcium supplementation was discontinued when the serum iPTH level reached the normal range. Hypoparathyroidism was retrospectively diagnosed as transient if the serum iPTH value on POD 1 was below the normal range but recovered to the normal range during the 2-year follow-up period. Permanent hypoparathyroidism was defined as a subnormal serum iPTH level that had persisted until the final follow-up examination reviewed in the current study.

### 3.3. Statistical Analysis

Quantitative data were reported as the means. The statistical analysis was performed by Statistical Analysis System JMP software version 8.0 (SAS Institute Japan, Tokyo, Japan). The differences were evaluated for statistical significance by the Pearson’s chi-square test. The significance level chosen was P < 0.05.

## 4. Results

Thyroidectomy had been performed in 296 patients (235 females and 61 males). TT had been performed in 36 of them and TT plus LN dissection in the other 260. Patients` characteristics were indicated in Table 1. The patients’ median age at the time of surgery was 54 years (range: 14-86). All patients were followed up. The median follow-up period was seven months (range: 7-20). On POD 1, 73 patients were hypocalcemic.

**Table 1:** Characteristics of Patients

	Extent of Surgery	
	TT a	TT a+ LN a Dissection
Gender F/M, No	25/11	210/50
Age, Y	53 ± 15	53 ± 13
Pathology, No		
	AG a 23	0
	FA a 4	0
	FTC a 7	7
	PTC a 2	253

^a^Abbreviations: TT, Total thyroidectomy; LN, lymph node; AG, Adenomatous goiter; FA, Follicular adenoma; FTC, Follicular cancer; PTC, Papillary cancer

**Figure 1:**
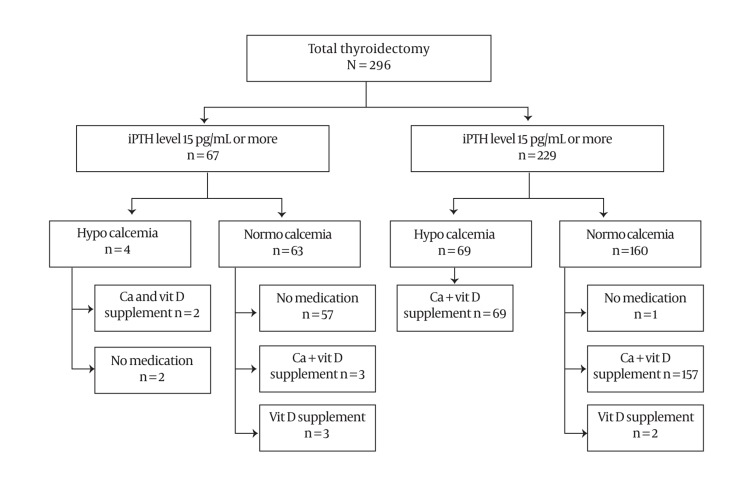
Flow Chart of the Patient Population Investigated on Postoperative Day 1. iPTH, Intact Parathyroid Hormone; Vit D, Vitamin D

The serum iPTH value on POD 1 was within the normal range in 67 of the 296 patients (Figure 1). The serum iPTH level and calcium level were within the normal range in 63 patients, and six of them received vitamin D and/or calcium supplementation in order to prevent hypocalcemia under doctor’s judgment. One parathyroid gland had been preserved in situ in 13 of the 63 eucalcemic patients with normal parathyroid function, two had been preserved in situ in 35 of them, three in seven of them, and four in eight of them. Postoperative hypocalcemia developed in four of the 67 patients with a normal iPTH value on POD 1. Two of the four patients with postoperative hypocalcemia did not receive any supplementation because their hypocalcemia was mild and they were asymptomatic. The serum iPTH was below the normal range in 229 patients on POD 1. Postoperative hypocalcemia developed in 69 of these 229 patients on POD 1, and calcium supplementation and vitamin D supplementation were started. The serum calcium value on POD 1 was within the normal range in 160 of the 229 patients with postoperative hypoparathyroidism. One of these 160 patients did not require oral calcium supplementation to maintain normocalcemia, but 157 of them received calcium and vitamin D supplementation. Serum iPTH value sensitivity on POD 1 to predict hypocalcemia was 94.5%, and serum iPTH value specificity on POD 1 to predict hypocalcemia was 28.3%.

None of the parathyroid glands were preserved in situ in 41 patients, all of them developed hypoparathyroidism on POD 1. One parathyroid gland was preserved in situ in 106 patients, 91 of them developed hypoparathyroidism on POD 1 (86%). Two parathyroid glands were preserved in situ in 128 patients, 91 of them developed hypoparathyroidism on POD 1 (71%). Three parathyroid glands were preserved in situ in 11 patients, four of them developed hypoparathyroidism on POD 1 (36%). And four parathyroid glands were preserved in situ in ten patients, two of them developed hypoparathyroidism on POD 1 (20%). The incidence of hypoparathyroidism on POD 1 was significantly higher in the group with smaller number of preserved parathyroid glands in situ (P < 0.01).

During the follow-up period the serum iPTH level of 192 out of a total 229 patients who developed hypoparathyroidism on POD 1 recovered to the normal range. The median duration of the hypoparathyroidism was three months. In 52 of them parathyroid function recovered within one month after surgery. Their average serum iPTH value on POD 1 was 7.6 pg/mL. The average iPTH value on POD 1 of the 192 patients with transient hypoparathyroidism was 6.7 pg/mL. Thirty-seven patients developed permanent hypoparathyroidism, and their average serum iPTH value on POD1 was 4.9 pg/mL. There was a significant difference between the serum iPTH values on POD 1 of the transient and the permanent hypoparathyroidism groups (P < 0.01).

The incidence of hypoparathyroidism on POD 1 was higher in the TT plus LN dissection group than in the TT group (Table 2). The average serum iPTH value on POD 1 was 15.1 pg/mL in the group who had undergone TT alone versus 8.8 pg/mL in the group undergone TT plus LN dissection (P < 0.01). Permanent hypoparathyroidism developed in six TT patients and in 31 TT plus LN dissection patients. The incidence of permanent hypoparathyroidism was significantly higher in the TT plus LN dissection group than in the TT group. None of the parathyroid grafts that had been successfully implanted in the patients with permanent hypoparathyroidism ceased to function later. The incidence of permanent hypoparathyroidism was significantly higher in the smaller numbers of present (preserved + autotransplanted) parathyroid glands (P < 0.01). Two patients ,whose all four parathyroid glands had been preserved in situ developed permanent postoperative hypoparathyroidism (their serum iPTH values on POD 1 were 5.3 pg/mL and 11.3 pg/mL, respectively), but their serum iPTH levels gradually increased to 13.1 pg/mL and 12.5 pg/mL, respectively, by 11 months after TT, and vitamin D supplementation had been continued in both patients.

**Table 2:** Incidence of Hypoparathyroidism on Postoperative Day 1

	No. of Patients With Hypoparathyroidism/Total No. of Patients in the Group(%)
TT ^a^	14/36 (39%)
TT ^a^+ LN ^a^dissection	215/260 (83%)

^a^Abbreviations:TT;Total thyroidectomy; LN, Lymph node

There was no correlation between the time required for the low iPTH values on POD 1 to recover to within the normal range and the number of present parathyroid glands in the patients with transient hypoparathyroidism. The average periods of serum iPTH level rising to the normal range after the surgery were five months in the autotransplanted parathyroid gland group and three months in the two autotransplanted parathyroid gland groups, the three autotransplanted parathyroid gland group, and the four autotransplanted parathyroid gland group (P = 0.58).

## 5. Discussion

There are several potential causes for hypocalcemia after thyroidectomy, this complication is caused by a inadvertent parathyroidectomy (1, 2). The number of parathyroid glands remaining in situ after TT is of crucial importance to maintaining calcium homeostasis in the immediate postoperative period. Parathyroid autotransplantation for removed and devascularized parathyroid glands is a simple method to reduce postoperative hypoparathyroidism incidence, and is also a useful method to avoid protracting hypocalcemia after total thyroidectomy (5).

The TT procedure to prevent postoperative hypoparathyroidism combines parathyroid gland preservation in situ with autotransplantation of resected or devascularized parathyroid glands (4). It is easy for the surgeon to identify the superior parathyroid glands and preserve their blood supply intact. In most TT plus LN dissection cases in the current study the inferior parathyroid glands were removed with the surrounding adipose tissue, and it is difficult to distinguish the glands from the surrounding swollen LNs. Surgeons need to identify the parathyroid glands in the excised adipose tissue. Sometimes the surgeons removed parathyroid glands unintentionally, and the parathyroid glands were discovered during the pathological examinations. In this study, the number of parathyroid glands that were preserved in situ and autotransplanted were counted from the records of the surgical procedures and that of the pathological examinations. Unintentional parathyroidectomy and parathyroid autografting were found to be associated with a higher risk of hypocalcemia (1). Surgical training and expertise in how to preserve parathyroid gland function are required to prevent postoperative hypoparathyroidism after TT plus LN dissection.

The results of this study showed that the serum iPTH value on POD 1 was a very sensitive predictor of postoperative hypocalcemia. A previous study reported finding that that the post-thyroidecomy serum iPTH level predicted hypocalcemia (6). In the current study four of the 67 patients whose serum iPTH value on POD 1 was within the normal range developed hypocalcemia. The data showed the high sensitivity of the serum iPTH value on POD 1 to predict hypocalcemia. A combined measurement of iPTH and serum calcium levels is recommended to identify patients at risk for developing hypocalcemia. Severe, progressive hypocalcemia is unlikely with a normal iPTH level, and thus iPTH can be used cautiously to facilitate early discharge for many patients (6).

The serum iPTH levels of the patients who developed postoperative hypoparathyroidism were below the normal range on POD 1, and their serum iPTH levels gradually increased thereafter. Parathyroid function recovered within the first month after surgery in 20% of the patients with postoperative hypoparathyroidism in the current study series. The previous study reported that serum iPTH levels recovered within 6 months after surgery, and they stabilized at almost the same level (7). At 1 year after surgery the serum iPTH recovery ratio in the parathyroid gland preservation in situ group was higher than the autotoransplantation group. The serum iPTH levels of the patients whose parathyroid glands were preserved after TT and the patients with preserved parathyroid glands whose removed parathyroid glands were autotransplanted after TT, recovered to their preoperative level. TT with parathyroid autotransplantation alone induced inadequate recovery of parathyroid function (7). None of the patients in the present study developed late hypoparathyroidism. Recovery of the serum iPTH levels of seven hypoparathyroidism patients to the normal range was observed after more than 1 year postoperative follow-up. The results of the current study suggest that the serum iPTH value on POD 1 after TT is a useful permanent hypoparathyroidism development predictor.

A phenomenon in which the serum iPTH levels rise to values above the normal range during the postoperative period has been reported in Graves’ disease patients (8). Patients with Graves’ disease, patients who had undergone a reoperation, and patients with a coexisting parathyroid adenoma were excluded from subjects of this study, because of the altered calcium homeostasis. No such changes in postoperative serum iPTH levels were observed in the thyroid nodule patients in this study.

Routine prescription of oral calcium and/or vitamin D supplements has been advocated by some endocrine surgeons to minimize the incidence of hypocalcemia and also to shorten the patients’ hospital stays. Routine use of oral calcium supplements not only reduces the incidence of hypocalcemia but also its severity, and it is not associated rebound hypercalcemia problem that sometimes occurs during routine combined use with vitamin D supplements (6).

Serum iPTH level after surgery predicts hypocalcemia after TT or TT plus LN dissection. Serum iPTH measurement on POD 1 is useful to predict postoperative hypocalcemia, and to determine when to start oral calcium and vitamin D supplementation for patients who developed postoperative hypoparathyroid to maintain normocalcemia. A single serum iPTH measurement on POD 1 can be used as the basis to decide whether or not to institute early calcium and vitamin D supplementation to prevent and reduce the severity of postoperative hypocalcemia.

## References

[1] Escobar-Jimenez F, Torres EV, Picon A, Megias M, Moll J (1977). Hypocalcaemia and thyroid surgery.. Lancet..

[2] Pattou F, Combemale F, Fabre S, Carnaille B, Decoulx M, Wemeau JL (1998). Hypocalcemia following thyroid surgery: incidence and prediction of outcome.. World J Surg..

[3] Lombardi CP, Raffaelli M, Princi P, Santini S, Boscherini M, De Crea C (2004). Early prediction of postthyroidectomy hypocalcemia by one single iPTH measurement.. Surgery..

[4] Kihara M, Yokomise H, Miyauchi A, Matsusaka K (2000). Recovery of parathyroid function after total thyroidectomy.. Surg Today..

[5] Katz AD (1981). Parathyroid autotransplantation in patients with parathyroid disease and total thyroidectomy. Indications in 117 cases.. Am J Surg..

[6] Grodski S, Serpell J (2008). Evidence for the role of perioperative PTH measurement after total thyroidectomy as a predictor of hypocalcemia.. World J Surg..

[7] Kihara M, Miyauchi A, Kontani K, Yamauchi A, Yokomise H (2005). Recovery of parathyroid function after total thyroidectomy: long-term follow-up study.. ANZ J Surg..

[8] Yano Y, Nagahama M, Sugino K, Ito K (2008). Long-term changes in parathyroid function after subtotal thyroidectomy for graves’ disease.. World J Surg..

